# Deep Learning-Based Feature Extraction with MRI Data in Neuroimaging Genetics for Alzheimer’s Disease

**DOI:** 10.3390/genes14030626

**Published:** 2023-03-01

**Authors:** Dipnil Chakraborty, Zhong Zhuang, Haoran Xue, Mark B. Fiecas, Xiatong Shen, Wei Pan

**Affiliations:** 1 Division of Biostatistics, School of Public Health, University of Minnesota, Minneapolis, MN 55455, USA; 2 Department of Electrical and Computer Engineering, University of Minnesota, Minneapolis, MN 55455, USA; 3 School of Statistics, University of Minnesota, Minneapolis, MN 55455, USA

**Keywords:** CNNs, endophenotypes, genome-wide association study (GWAS), principle components (PCs), SNPs

## Abstract

The prognosis and treatment of patients suffering from Alzheimer’s disease (AD) have been among the most important and challenging problems over the last few decades. To better understand the mechanism of AD, it is of great interest to identify genetic variants associated with brain atrophy. Commonly, in these analyses, neuroimaging features are extracted based on one of many possible brain atlases with FreeSurf and other popular software; this, however, may cause the loss of important information due to our incomplete knowledge about brain function embedded in these suboptimal atlases. To address the issue, we propose convolutional neural network (CNN) models applied to three-dimensional MRI data for the whole brain or multiple, divided brain regions to perform completely data-driven and automatic feature extraction. These image-derived features are then used as endophenotypes in genome-wide association studies (GWASs) to identify associated genetic variants. When we applied this method to ADNI data, we identified several associated SNPs that have been previously shown to be related to several neurodegenerative/mental disorders, such as AD, depression, and schizophrenia.

## 1. Introduction

Alzheimer’s disease (AD) is an irreversible neurodegenerative disease that causes a decay in brain tissue, resulting in a loss of brain functions. It is the most common cause of dementia, affecting roughly 5.8 million Americans aged 65 or above. AD has been identified as the sixth largest cause of death in the US [1,2]. The approximate cost of managing relevant finances of patients with Alzheimer’s disease was USD 277 billion in 2018 (Alzheimer’s Association, 2018), which is expected to almost double up in the next 20 years. There is no existing treatment to cure the disease. Unraveling the genetic architecture of AD may help to develop new preventive and treatment strategies.

Alzheimer’s Disease Neuroimaging Initiative (ADNI) [3] is an ongoing research project on the development and progress of the disease. Magnetic resonance imaging (MRI) technologies are used to measure brain functions and structures and to assess the genetic architecture of brain-related diseases using imaging-derived endophenotypes [4]. Data from these scans have been used to evaluate the neurological and psychiatric distortions created by diseases such as Parkinson’s disease, Alzheimer’s disease, schizophrenia, autism, and dementia. ADNI has provided a unique opportunity to combine data on MRI imaging and genetics.

A crucial challenge in this kind of study is to represent medical images as useful information that can ease clinical decision making [5]. Traditionally, the most used approach has been image-on-scalar regression using mass univariate analysis (MUA). This method uses a general linear model at each voxel and calculates a voxel-wise test statistic. One drawback of the approach is that spatial correlation is not considered here, which may result in low power. One of the potential remedies is to use all the voxels collectively as a tensor. Pan et al. (2019) [6] proposed a penalized approach to select subsets from tensors after adjusting for the other covariates. In Miranda et al. [7], tensor partition regression modeling (TPRM) was used to predict disease status using structural medical resonance imaging (sMRI) data. Authors Shi and Kang (2015) [8] modeled spatially varying functions as thresholded, multiscale, Gaussian processes from a Bayesian point of view. In Feng et al. (2019) [9], the authors developed a Bayesian scalar-on-image regression model to combine the highly dimensional image data and clinical data to predict different outcome states. The primary concern is that most of these statistical methods lose efficiency because of the high dimensionality of the array images as well as the complex structure. Advances in neuroimaging, deep learning, and genetics have opened new directions for feature extraction as endophenotypes to study the influence of genetics on brain structure and functions [10,11]. In [12], the authors proposed a classifier ensemble combining convolutional neural networks (CNNs) and ensemble learning (EL) to obtain robust learning performance. In [13], the authors showed a comparative result of different types of deep learning methods using MRI data from Alzheimer’s patients, whereas the authors of [14] used greedy-score-based feature selection based on MRI images to discriminate among AD subjects; however, there are limited discussions based on combining image data and biomarker data, which is the primary contribution of this work.

A common approach to medical imaging analysis is to use 2D convolutional neural networks (CNNs). While two-dimensional CNNs have advantages in terms of data handling and limited GPU usage during optimization, often, researchers only use one of the sagittal, transverse, and coronal slices of the three-dimensional MR images, and the lack of knowledge about how to extract complete information using 2D patches prevents researchers from learning many sorts of important and robust information. In recent times, research has shown that improved results are achievable when using full volumetric data. In Zhao et al. (2021) [15], the authors used a Bayesian semi-parametric model based on ADNI data to jointly estimate voxel-specific heritability over whole-brain imaging traits. In Bao et al. (2021) [16], the authors performed an empirical study on amyloid imaging and whole-genome sequencing data using spatially connected voxels and showed higher estimated heritability measures. In this paper, we explored three-dimensional whole-brain structure MRI data obtained from the ADNI database to avoid losing any information.

In addition, in genome-wide association analyses, researchers have extracted features from images using FreeSurf and other advanced normalization tools to use as phenotypes. In Grasby et al. (2020) [17], the authors used the surface area and cortical thickness of the cortex and cortical regions in a genome-wide association meta-analysis to link several genetic variants with them. Zhao et al. (2020) [18] found significant genetic correlations between global functional connectivity measures and the volumes of several brain regions. In Zhao et al. [19], the authors used consistent standard procedures via advanced normalization tools on MRI data and produced one hundred one region-based and overall brain volume phenotypes, which were further used in GWAS analyses. Many researchers have also used the AD statuses as phenotypes to find genetic biomarkers [20]. Shen et al. [21] presents a detailed review of several such methods.

The work in this paper focuses on two important aspects: (i) developing a completely data-driven feature extraction approach to ensure that the extracted features are related to AD, which is accomplished with CNN-based classification of disease status, namely, cognitively normal (CN), mild cognitive impairment (MCI), and Alzheimer’s disease (AD); (ii) using these image-derived features as endophenotypes in a genome-wide association study (GWAS) to find out the genetic variants (SNPs) possibly related to AD. From the feature extraction point of view, it can be compared to the decomposed basis tensor layers mentioned in Tang et al. (2020) [22]. Section 2 and Section 3 describe the data and methods used in the proposed deep learning and genome-wide association studies. Section 4 and Section 5 contain the results of our study and relevant discussion, and Section 6 presents overall concluding remarks.

## 2. Data

In this paper, the imaging dataset as well as the genetic data were obtained from the Alzheimer’s Disease Neuroimaging Initiative (ADNI) website (http://adni.loni.usc.edu (accessed on 27 February 2023). ADNI was launched in 2003 by National Institute of Biomedical Imaging and Bioengineering (NIBIB), National Institute on Aging (NIA), thirteen pharmaceutical companies, and two other agencies, and was headed by Michael W. Weiner, MD, VA Medical Center and University of California, San Francisco. For most recent information and updates, see www.adni-info.org (accessed on 27 February 2023).

We first focused on the data containing the MRI scans of the 817 ADNI-1 subjects divided in three groups, i.e., 188 early Alzheimer’s disease (AD) patients, 400 subjects showing mild cognitive impairment (MCI), and 229 cognitively normal patients (CN), and for the GWASs, we considered the corresponding genetic data of 757 subjects.

### Preprocessing and Description

In ADNI-1, MRI data were obtained using different of MRI scanners with respect to particular protocols for each scanner. They included T1-weighted images that were obtained with volumetric three-dimensional sagittal MPRAGE or similar protocols using different resolutions [23]. The most commonly used image files were T1-weighted images obtained using 3D sagittal MPRAGE or other similar protocols. Magnetization-prepared rapid acquisition with gradient echo (MPRAGE) images from the LONI database underwent image processing steps including Gradwrap, B1 non-uniformity correction, and N3 correction. Gradwrap corrects image geometry distortion due to gradient non-linearity. B1 non-uniformity corrects intensity non-uniformity in the image. N3 is a histogram peak sharpening method that is applied to all images. Details on image preprocessing can be found at https://adni.loni.usc.edu/methods/mri-tool/mri-pre-processing/ (accessed on 27 February 2023).

**Figure 1:**
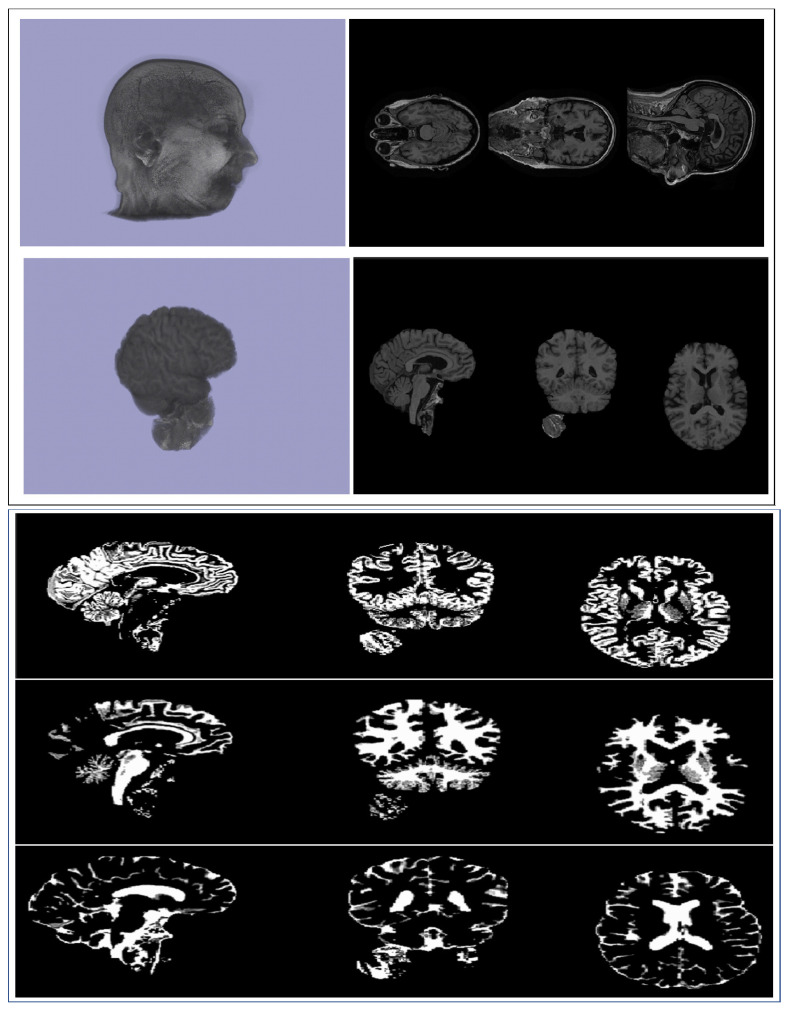
(**Top row**) An original MRI image and the corresponding axial views. (**Second row**) Brain image extracted using BET tool and its axial views. (**Bottom three rows**) Segmentation of the extracted brain image using FAST; from top to bottom: gray matter (GM), white matter (WM), and cerebrospinal fluid (CSF) images, respectively.

We used Brain Extraction Tools (BET) [24] to extract the usable brain images. BET is a part of the well-known FMRIB (Functional Magnetic Resonance Imaging of Brain) Software Library (FSL) [25], which contains tools for image and statistical analysis for functional, structural, and diffusion MRI brain imaging data. Using BET, we deleted non-tissue regions from an image of the whole-brain structure (Figure 1, Left). In the whole image, a large number of voxels are zero. This is expected, as the brain is in a more centrally enclosed part of the image. Considering this fact and to minimize the computational cost, we removed the encompassing regions with no information contained. Finally, an image acquisition matrix of dimensions 155 × 155 × 95 was used in further studies.

## 3. Methods

### 3.1. Classification and Feature Extraction

Using deep learning to detect and classify diseases has gained a lot of attention in recent years. Researchers from across the globe use convolutional neural networks (CNNs) for imaging as well as sequential [26] problems. Numerous studies in the last few years have focused on detecting Alzheimer’s disease and other types of dementias. The timely detection and diagnosis of Alzheimer’s disease help to control or slow down the progression of the disease. Researchers have used 3D CNN and its layers to understand the behavior of disease spread.

In Kruthika et al. [27], autoencoders were used to extract features from 3D patches, obtaining a better result than when using traditional 2D patches. In [28], the authors show that the performance of the classification problem in case of dermoscopic analyses significantly outperforms that of raw images. Researchers have also used LSTM (long short-term memory) layers on top of three-dimensional convolutional layers to improve the performance of models. Three-dimensional fully connected CNN layers were obtained for deep feature representations, and LSTM was applied on these features to improve the performance. In Gao et al. [29], the authors improved the accuracy of AD, lesion, and normal aging classification to 87.7% using seven-layer deep three-dimensional CNN on 285 volumetric CT head scans from Navy General hospital, China. In [30], the authors established how we can take advantage of brain imaging to define an auxiliary diagnostic process. In the literature of medical imaging, 3D CNN models have been proposed for the classification of 3D images, as they can fully utilize the context information. However, the development of 3D CNNs has been limited to simple architectures and small image sizes due to the high computational cost. In this section, we describe the 3D CNN architectures we used for classification and feature extraction. We developed 3D CNN models with different architectures and different depths to classify the study participants into three categories, namely, AD, MCI, and CN, and extracted the output from a fully connected layer of 100 vectors, known as neurons, to be used as automatically extracted features. To further use the extracted features as endophenotypes in GWASs, we reduced the dimension of the feature vectors using principal components analysis (PCA) and then used the top 10 PCs as endophenotypes.

#### 3.1.1. Whole-Brain Structure

**Figure 2:**
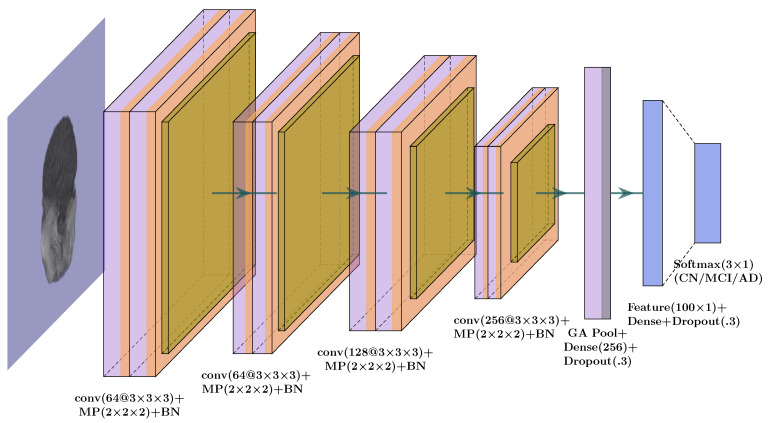
Convolution neural network architecture used for the whole-brain structure. From the penultimate dense layer, 100 neurons are extracted as the features.

We started the study by considering the extracted brain images obtained using BET. We used a 14-layer 3D convolutional neural network model (Figure 2) and added max pooling layers for dimension reduction and 3D convolution, as a natural adaption of 2D convolution. We also included batch normalization to each pooling layer. Throughout the CNN architecture, we used rectified linear units (ReLU) [31] as the default activation function, except for softmax [32] in the last layer for classification. In the proposed CNN model, we performed hyperparameter tuning to obtain the best possible results by modulating parameters such as kernel size, steps, number of channels, and dropout rate [33]. To minimize the over-fitting issue, we used two dropout layers with 30% probability.

From the CNN architecture, we extracted the output from the penultimate layer, a fully connected layer with 100 neurons, and used it as the input to a logistic regression model or support vector machine (SVM) for classification. Furthermore, we applied transfer learning algorithms. Transfer learning-based neural network models have recently gained popularity. With some necessary modifications, we implemented the idea of transfer learning models such as AlexNet, ResNet50, and VGG16 in our study.

AlexNet [34], a convolutional neural network, was designed by Alex Krizhevsky in collaboration with Ilya Sutskever and Geoffrey Hinton to compete and win the first place at the ImageNet Large Scale Visual Recognition (ILSVR) challenge. The Visual Geometry Group (VGG16) [35] convolutional neural network proposed by K. Simonyan and A. Zisserman secured second place at ILSVRC-2014 and made an improvement over AlexNet by using multiple 3 × 3 filters. Residual Network (ResNet) [36], based on a residual function, won the first prize at ILSVRC-2015. We used the three-dimensional counterparts of their models and applied a transfer learning technique by replacing the top layer of the network with our classification layer.

To avoid over-fitting in our neural network architectures, we opted for L2-norm regularization and implemented image augmentation by rotating the images by −20, −10, −5, 5, 10, and 20 degrees, respectively, i.e., for each input image in the training set, we had six more images. In the model architectures, ReLU layers following convolution layers were used to introduce the non-linearity necessary for class discrimination. The pooling layers were used to ensure that neurons did not learn redundant information and the model did not grow too large while achieving some location invariance. One of the primary concerns of the study was to find out genetic variants associated with AD; hence, we decided to build a binary classification model as well as one based on AD and CN subjects only.

#### 3.1.2. Multi-Branch CNN

**Figure 3:**
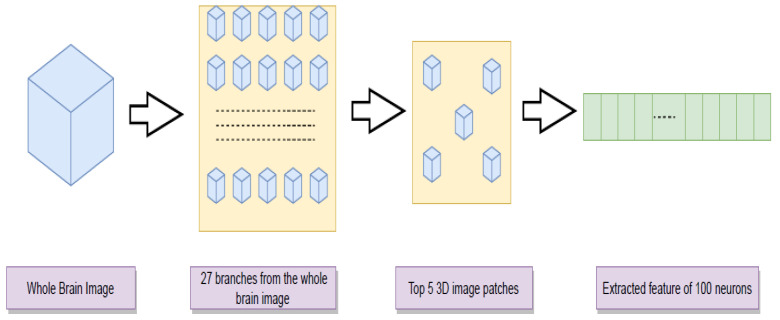
Pipeline of feature extraction using a multi-branch CNN model.

Using smaller parts of a 3D image often enables one to focus on the most locally relevant information in classification. In our study, we divided the whole-brain structure along the *x*-, *y*-, and *z*-axes in three parts, respectively, resulting in 27 smaller and over-lapping 3D image patches. We individually trained a separate 14-layer CNN architecture with each of these pieces. The validation accuracy of these smaller patches was found to be noticeably higher for some parts than the test accuracy of the whole-brain CNN model. Thus, we selected the top five CNN models based on the accuracy levels and used them to build the final CNN model for feature extraction (Figure 3). Similar to the whole-brain image scenario described above, we collected a layer of fully connected neurons and used PCA to obtain the top 10 PCs to be further used in the genome-wide association studies.

### 3.2. Segmentation

We applied FMRIB Automated Segmentation Tool (FAST) [37] in the FSL software package to obtain individual 3D images corresponding to three tissue types, namely, gray matter (GM), white matter (WM), and cerebrospinal fluid (CSF) (Figure 1, Right). Further, we carried out CNN-based feature extraction and then GWASs on these segmented images similar to those discussed for whole-brain and multi-branch structures.

### 3.3. GWAS

One of the most important risk factors of AD is a family history of dementia. Families with multiple members affected by AD are more susceptible to AD. Though we have made significant progress in research related to AD, perhaps, most AD-related genetic variants and genes are still unknown.

In this work, we applied PCA to choose the top 10 principal components (PCs) from the extracted feature vectors. Each PC was then used as an endophenotype to conduct GWASs (while adjusting for additional covariates such as age, sex, education, handedness, etc.). The findings of the GWAS corresponding to the most relevant CNN models have been discussed in the next section.

### 3.4. PC vs. Gray Matter Volume of Regions of Interests (ROIs)

A number of recent studies have shifted their focus to developing machine learning models that can be easily interpreted. In Ribeiro et al. [38], the authors discuss using explainable methods to be able to trust the predictions made by “black-box” models such as CNNs. In this work, we used linear regression to investigate the relations between the extracted principal components and some commonly used ROIs already available on the ADNI website, such as the GM volumes of left hippocampus (HIPPL), para left hippocampus (PARAHIPPL), cerebellum, etc., extracted from 1.5 T ADNI MPRAGE MRI segmented gray matter maps using longitudinal VBM (voxel-based morphometry). These ROIs have been previously shown to change significantly between AD and control subjects [39].

### 3.5. Identifying Genetic Pathways

Recent advancement in the scientific world has seen a broader spectrum being created for identifying genetic pathways and for the development of targeted therapies [40,41]. In this study, we worked with the principle components and the derived GWAS results to identify any affected pathways. We used Functional Mapping and Annotation of Genome-Wide Association Studies (FUMA GWAS (version 1.5.1)) [42] software to annotate and interpret the GWAS results obtained from the PCs. The SNP2GENE function in the software was used to input the GWAS summary results and to identify the leading SNPs. In a second step, we used the GENE2FUNC function to annotate the genes and possibly enriched pathways.

### 3.6. Evaluation Criteria

The receiver operating characteristic (ROC) [43] curve is obtained by plotting the true positive rate (TPR) against the false positive rate (FPR). The area under the ROC curve (AUC) is one of the most widely used metrics for classification. The training AUCs reported in the paper are based on validation sets. We used the AUCs obtained from the models to compare their prediction accuracy. We split the whole image dataset into training (80%), validation (10%), and test (10%) subsets and calculated the (test) AUC with the test subset; then, the above process was repeated 10 times, and we calculated the average (test) AUCs reported in this paper. The models with the highest prediction accuracy were used for the GWASs.

## 4. Results

### 4.1. Whole-Brain Structure

The training AUC of the CNN model corresponding to the images of the whole-brain structure was 0.88, and the test AUC was 0.72. By performing GWASs with each of the top 10 PCs as the phenotype yielded 149 SNPs at *p*-value $\leq 5\times 10^{-6}$, including rs2075650, which has been found to be consistently associated with AD [20,44]. The Apolipoprotein E (APOE) gene and the TOMM40-APOE locus, tagged by rs2075650 in the translocase of outer mitochondrial membrane 40 (TOMM40) gene, are both considered to be responsible for AD. TOMM40 rs2075650 (chromosome 19q13.32) has been one of the most important SNPs found to be associated with AD. TOMM40 encodes the translocase of the mitochondrial outer membrane (TOM) complex through mitochondrial channel protein TOM40 [45]. Changes in the mitochondrial metabolism have been accepted as a feature in patients with Alzheimer’s disease. The eMERGE network showed an association between rs2075650 and AD using electronic medical records [44].

Next, we implemented logistic regression considering the extracted feature vectors as inputs for corresponding subjects, and the model produced a training AUC of 0.78 and a test AUC of 0.59. Although the last two layers of the CNN were similar to logistic regression, due to L2/ridge-type regularization (i.e., weight decay) in the former, while the dimension of the features (i.e., 100) was high relative to the sample size in a few hundreds, there was a substantial performance difference between the two. Similarly, support vector machine (SVM) yielded training and test AUCs of 0.79 and 0.61, respectively. CNN models based on transfer learning, such as AlexNet, ResNet50, and VGG16, produced a training AUC within the range of 0.84–0.87 and a test AUC of 0.69–0.72. None of these models provided improved accuracy measures in the classification problem and thus were dropped in future consideration for feature extraction and GWASs.

Over-fitting and small sample sizes are two of the main growing concerns regarding neural network models, especially in medical image data analysis. We trained the CNN model with augmentation by rotating the images by six different angles. Introducing the augmentation of the training sample made the input richer with information and improved the AUC measure of the test dataset up to 0.75. The GWAS based on the features derived from this model yielded 3 SNPs at *p*-values $\leq 5\times 10^{-8}$, including rs2075650, rs1158059 [46], and rs823955 [47], and 35 SNPs at the marginal significance level of $5\times 10^{-6}$. This shows that our method works better than existing methods based on ROIs, such as FreeSurfer or voxel-based morphometry (VBM) [48,49]. In [49], the authors presented a table comparing the *p*-values of several SNPs tested on a set of univariate traits.

**Figure 4:**
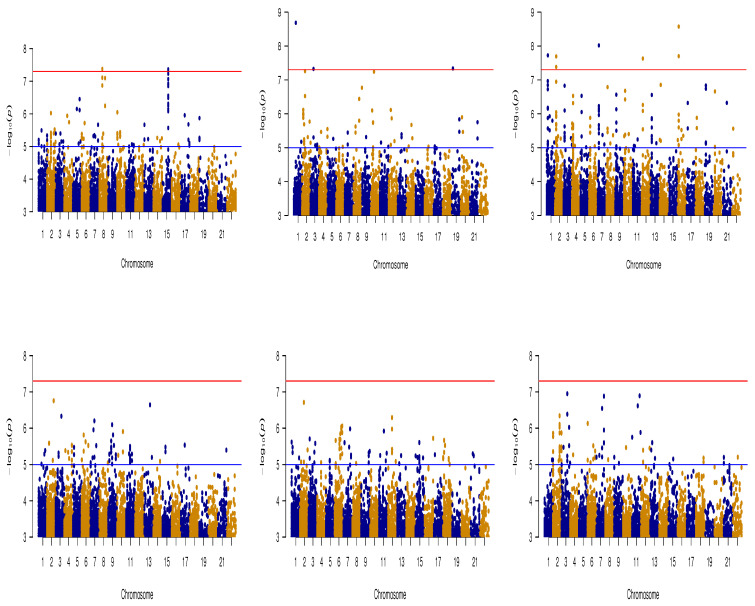
Manhattan plots. (**Top panels**) The left panel corresponds to binary classification of AD vs. CN; the panel in the middle is for whole-brain structure with augmentation; and the right panel is for the multi-branch model based on the whole-brain structure. (**Bottom panels**) The left panel is for the multi-branch model based on GM images; the middle panel is for the multi-branch model based on WM images; and the right panel is for the multi-branch model based on CSF images.

Many research studies have been conducted with the primary focus on identifying the genetic differences between AD and CN subjects. For that purpose, we used a CNN model to address this binary classification problem, which yielded a training AUC of 0.96 and a test AUC of 0.90. A genome-wide association scan produced 2 SNPs at the p-value significance level of $5\times 10^{-8}$ and 53 SNPs at $5\times 10^{-6}$, with the inclusion of rs7836628 ($4.21\times 10^{-8}$) and rs12708438 ($4.33\times 10^{-8}$) SNPs. This list of SNPs also includes rs2196315 (chr8:130787049) ($8.02\times 10^{-8}$), corresponding to gene ADCY8 (Intron Variant), which has been found to be connected with dissociative amnesia (corresponding to rs726411, located in the 8q24.22 region [50], and rs263238 [51] with $p=2.40\times 10^{-6}$ ), whereas SNP rs2395095 of the Adenosine Kinase (ADK) gene has been previously found to be responsible for triggering cognitive impairment and seizures [52,53]. The Manhattan plot on the top-left panel of Figure 4, corresponding to the AD vs. CN classification, shows a large number of significant SNPs on chromosome 15 (chr15:29176591, chr15:29175402, chr15:29190239, and chr15:29225416), which has been an increasing focus in the field of neurology and has shown associations with several neurodegenerative/mental/brain diseases, such as dyslexia, autism, ring 15 chromosome syndrome, Angelman syndrome, and hyperlexia [54]. Table 1 summarizes the most significant SNPs corresponding to the principal components obtained with the discussed models along with their chromosomal positions and *p*-values from the relevant GWASs.

We separately used the CNN model discussed in the whole-brain section for the GM, WM, and CSF segments. The training and test AUCs of the CNN models in case of GM images were found to be 0.82 and 0.70, respectively. Similarly, the training and testing measures of AUC for WM images were 0.88 and 0.68, and those for CSF images were 0.77 and 0.54, respectively. The relevant GWASs did not identify any significant SNPs at a *p*-value of $5\times 10^{-8}$.

### 4.2. Multi-Branch CNN

For the multi-branch CNN, we trained a separate CNN model for each of the 27 sub-parts of 3D image patches. We selected the top five patches and combined them together to obtain our final model. The training accuracy obtained for the final model was 0.86, and the testing accuracy thus obtained was 0.76. The feature vector was extracted from the final model to obtain the top 10 principal components. The GWAS conducted with these PCs identified 8 SNPs at *p*-value $5\times 10^{-8}$ and 87 SNPs at a *p*-value of $5\times 10^{-6}$. The list of significant SNPs includes rs1397645 (ch4:121036579), corresponding to Neuron Derived Neurotrophic Factor (NDNF), which helps to protect and culture hippocampal neurons against amyloid beta-peptide toxicity [55]. Next, we used the multi-branch modeling technique on GM images. The training and test AUC measures obtained were 0.84 and 0.74. With the GWAS study, we identified that 35 SNPs were significant at a *p*-value of $5\times 10^{-6}$. This list of SNPs includes rs173754 ($3.33\times 10^{-6}$), which has been shown to be responsible for ADHD (attention deficit hyperactivity disorder).

This list of SNPs includes rs9257694 (chr6:29306709) ($1.55\times 10^{-6}$), which is associated with OR14J1 (olfactory receptor family 14 subfamily J member 1). Though no concrete conclusions have been drawn, abnormalities and impaired functions of the olfactory system have been reported in AD, and these symptoms have been seen to appear earlier than many other symptoms. Table 2 gives us a glimpse of the most significant SNPs corresponding to the principal components obtained with the multi-branch models, along with their chromosomal position and calculated *p*-values from the relevant GWAS studies.

The multi-branch model based on the WM images produced AUC measures of 0.82 and 0.70 on the training and test datasets, respectively. The GWAS based on this model identified 35 SNPs (Figure 4, bottom panel on the left) at the marginal significance level of $5\times 10^{-6}$. On the other hand, the model based on the CSF images produced AUCs of 0.77 on the training data and 0.66 on the test data, and a further GWAS analysis yielded 21 SNPs, but the SNPs were not found to be associated with any well-known gene. The list of significant SNPs includes rs11710427, rs15820, rs9791663, rs9791663, rs3132030, rs10880942, etc. The bottom panels of Figure 4 show the Manhattan plots for all the segment-wise multi-branch models. The Q-Q plots corresponding to all the six Manhattan plots show the corresponding *p*-values well calibrated with the genomic inflation factor (lambda) values, all very close to 1 (within a range of 0.989 to 1.033). The plots are included in the Supplementary Materials.

### 4.3. PC vs. Gray Matter Volumes of ROIs

**Figure 5:**
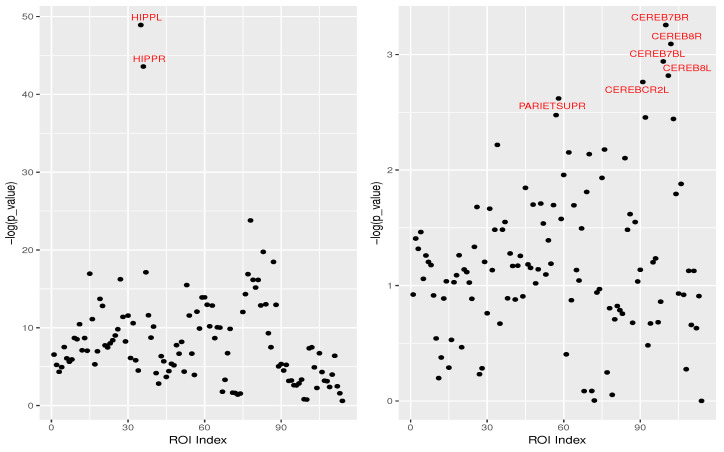
Marginal associations between each of the brain ROIs and one principal component. The *x*-axis shows the different ROI indices, and the *y*-axis shows the −log(*p*_value) obtained from the regression. The left panel is for PC2, and the right one is for PC3, with both having been obtained with whole-brain augmentation.

To enhance the interpretation of the extracted features, we investigated the relation between the mean gray matter volumes of brain ROIs and principal components using linear regression. Figure 5 shows for the second and third principal components obtained from the whole-brain structure with augmentation. The x-axis shows the ROI indices, and the *y*-axis shows the −log(*p*_value) obtained from the regression. The results demonstrate that the mean gray matter volumes of the ROIs, such as left/right hippocampus (HIPPL/R), right parietal superior (PARIETSUPR), and several parts of the cerebellum (left/right hemispheric lobule VIIb, CEREB7BL/R; left/right hemispheric lobule VIII, CEREB8L/R), show a higher level of significance than the other regions. However, using the principal components, we found SNPs that were not identified by only using the ROIs. One of the reasons may be that apart from the major effect of one brain ROI, the principal components may also have information on other brain regions. For instance, the third PC of the augmented model was found to be associated with rs1158059, corresponding to gene SNAP91, which has also been found to be involved in AD pathways [46]; the seventh principal component of the multi-branch model with the whole-brain method identified SNP rs10514441, related to gene WWOX, which plays an important role in AD through interactions with its protein partners, and cell pathology and degeneration [56]. These brain regions [57,58] have been previously shown to be associated with AD. Moreover, SNP rs2075650, associated with the second principal component (Table 1), has been shown to be strongly associated with the hippocampal volume of the brain [20,48]. The relations between the principal components and the available gray matter volumes of the ROIs give credibility to the used models and enhance the interpretability of the models. See Supplementary Materials for more plots.

### 4.4. Genetic Pathway Identification with Enrichment Analysis

We followed the two-step method described in FUMA GWAS software using the SNP2GENE and GENE2FUNC functions. For illustration purposes, we present the results from only one principle component (the seventh one) from the multi-branch model in this section. From a total of 511062 SNPs, 265 candidate SNPs and 7 lead SNPs were identified. These SNPs were mapped to seven independent genomic loci and were further mapped to five genes. Further, we used the GENE2FUNC function and found that two genes from chromosome 16 are related to traits such as adolescent idiopathic scoliosis [59] and total PHF-tau (SNP × SNP interaction) [60]. Due to the small sample size, we could not identify a sufficient number of even marginally significant SNPs and thus genes to detect enriched pathways.

## 5. Discussion

In this paper, we explored completely data-driven and automated feature extraction and used these features as phenotypes in the corresponding GWASs primarily based on two techniques: the first corresponds to CNN models trained on whole images, and the second, to multi-branch CNN models trained on divided sub-parts of whole images. We noticed that the initial CNN model based on the whole-brain structure was not the best-performing model in terms of prediction accuracy. The follow-up GWASs on the model also failed to capture significant SNPs at lower p-value cut-offs. One problem that the model suffered from was over-fitting. To tackle the issue, we implemented an augmentation method and performed the same steps on the augmented set of images, producing much more accurate results, along with significant SNPs (Table 1). Next, we adopted the binary classification problem between AD and CN subjects, instead of the existing tertiary classification problem. The used model, as expected, performed better, reaching up to 0.90 AUC measure on the test dataset. The relevant GWASs revealed significant SNPs at the usual genome-wide significance level of *p*-value $\leq 5\times 10^{-8}$ (Table 1).

The most important contribution of the paper is perhaps the idea of multi-branch CNN models, whereby we separately trained convolutional neural network models on 27 sub-parts of each whole-brain image and identified the top five patches with the highest validation accuracy to be included in the final model. Focusing only on the pertinent patches of the images helps us not only to localize relevant brain regions but also retrieve maximum local information, thus boosting the AUC measures of the models. Features pulled out from these models also pointed out more significant SNPs than their whole-image counterparts (Table 2).

Using the proposed machine learning method, we assessed the importance of brain image voxels in light of classification, thus allowing patches crucially associated with AD to be identified. Cumulatively, this study shows that based on sMRI data, an advanced machine-based neuroimaging approach can provide improved accuracy in AD detection and can also help to identify AD-related biomarkers.

Alzheimer’s disease has been one of the primary focuses of discussion for over a decade now because of its complex etiology. The failure of the “one-drug-for-all” idea has turned the attention of the scientific community towards targeted therapies. We expect that the SNPs (or genes) identified in this study can pave a path forward to guide this exploration in a positive direction.

## 6. Conclusions

Alzheimer’s disease is the most common form of dementia. The worldwide number of patients suffering from this disease is increasing everyday. The disease starts to develop years before the first noticeable symptoms start to appear. The study in this paper focuses on two major issues related to AD. Firstly, it proposes a method of automated feature extraction, compared with focusing on some pre-defined ROIs using existing software, through the classification of AD patients. It shows that using multi-branch CNN models, instead of the model for regular whole-brain structures, provides an increase in prediction accuracy and thus should be considered as an option for future brain-related research. It could be worthwhile to look into additional comparable datasets so that the underlying CNN algorithm can learn from more data. Secondly, the study identified the genetic variants related to AD. The work links the associated SNPs from the available genetic data to the imaging data of the brain. Some of the SNPs identified in our work have been previously shown to be related to mental/brain disorders, including schizophrenia, depression, and dementia.

## Figures and Tables

**Table 1:** Models based on whole-brain images with test AUCs (95% confidence intervals (CIs)) as well as contributing principal components and significant SNPs with their chromosomal positions and *p*-values. Novel and known SNPs are indicated by N and K, respectively.

Model	Test AUC (CI)	PC	SNP (N/K)	Chr	*p*-Value
ine Augmentation with respect to whole brain	0.75 (0.717, 0.782)	3	rs1158059 (K) [46]	1	2.06 $\times \;10^{-9}$
		2	rs2075650 (K) ([44])	19	4.58 $\times \;10^{-8}$
		4	rs823955 (N)	3	$4.72\times \;10^{-8}$
ine AD vs. CN binary classification	0.90 (0.885, 0.914)	4	rs7836628 (K) [47]	8	4.21 $\times \;10^{-8}$
		7	rs12708438 (N)	15	4.33 $\times \;10^{-8}$

**Table 2:** Multi-branch models with test AUCs (95% confidence intervals (CIs)) as well as contributing principal components and significant SNPs with their chromosomal positions and *p*-values. Novel and known SNPs are indicated by N and K, respectively.

Model	Test AUC (CI)	PC	SNP (N/K)	Chr	*p*-Value
ine Multi-branch	0.76 (0.729, 0.790)	4	rs1397645 (K) [55]	8	9.40 $\times \;10^{-8}$
		7	rs10514441 (K) [56]	16	2.67 $\times \;10^{-9}$
		9	rs17148126 (N)	7	9.59 $\times \;10^{-9}$
ine Multi-branch on GM images	0.74 (0.707, 0.772)	2	rs9257694 (N)	6	$1.55\times \;10^{-6}$
		3	rs173754 (N)	5	$3.33\times \;10^{-6}$

## Data Availability

The imaging dataset as well as the genetic data used in this work can be obtained from the Alzheimer’s Disease Neuroimaging Initiative (ADNI) website (http://adni.loni.usc.edu) (accessed on 27 February 2023). Codes supporting this study are available at https://github.com/Dipnil07 (accessed on 27 February 2023). The codes were implemented using Python, R, and Plink software.

## Supplementary Materials

The following supporting information can be downloaded at: https://github.com/Dipnil07 (accessed on 1 February 2023).
